# Mitotic centromere-associated kinesin is a novel marker for prognosis and lymph node metastasis in colorectal cancer

**DOI:** 10.1038/sj.bjc.6604379

**Published:** 2008-05-27

**Authors:** K Ishikawa, Y Kamohara, F Tanaka, N Haraguchi, K Mimori, H Inoue, M Mori

**Affiliations:** 1 Department of Molecular and Surgical Oncology, Medical Institute of Bioregulation, Kyushu University, 4546 Tsurumibaru, Beppu 874-0838, Japan; 2 Core Research for Evolutional Science and Technology (CREST), Japan Science and Technology Agency (JST), 4-1-8 Honcho Kawaguchi, Saitama, Japan

**Keywords:** MCAK, colorectal cancer, prognosis, lymph node metastasis

## Abstract

Mitotic centromere-associated kinesin (MCAK) is a microtubule depolymerase that is essential for proper kinetochore–microtubule attachment during spindle formation. Overexpression of MCAK has been correlated with aggressive forms of carcinoma, resulting in poor prognosis of colorectal cancer. The purpose of this study was to quantify *MCAK* expression in malignant and benign colorectal tissues and to determine if *MCAK* expression levels correlate with clinicopathologic factors and prognosis in colorectal cancer patients. Paired colorectal tissue samples from tumours and the corresponding normal tissues were obtained from 120 patients with colorectal cancer who underwent surgical resection. The real-time reverse transcriptase-PCR and immunohistochemistry were used to analyse mRNA and protein expression status with respect to various clinicopathological factors. *MCAK* expression was higher in colorectal cancer tissue (*P*<0.01) than in corresponding normal tissue, and this elevated expression level was markedly associated with factors such as lymph node metastasis (*P*=0.0023), venous invasion (*P*=0.019), peritoneal dissemination (*P*=0.021) and Dukes' classification (*P*=0.0023). Patients with high *MCAK* mRNA expression also showed a far poorer survival rate than those with low *MCAK* mRNA expression (*P*<0.01). Elevated *MCAK* expression was an independent predictor of overall survival and lymph node metastasis. These data suggest that *MCAK* expression may serve as a good marker of prognosis and lymph node metastasis in colorectal cancer.

Colorectal cancer is one of the most prevalent cancers in the world, accounting for nearly 10% of all cancer cases (Parkin *et al*, 2001). In Japan, the incidence of colorectal cancer has significantly increased during the past 50 years, in concert with changes in eating habits, other lifestyle parameters and longevity (Kono, 1996). Colorectal cancer is now the second most important cause of death from neoplastic disease in Japan (Tsukuma *et al*, 2004). Identification of the genes responsible for the development and progression of colorectal cancer is critical if improvements are to be made in the diagnosis and treatment of the disease.

Chromosomal instability, a recognisable feature of human cancers, is caused by continuous chromosomal missegregation during mitosis (Tomonaga *et al*, 2005). A physical connection (i.e., a kinetochore) between the spindle microtubules (MTs) and centromeric DNA is essential for appropriate chromosomal segregation (Tomonaga *et al*, 2005). During spindle formation, mitotic centromere-associated kinesin (MCAK), a microtubule depolymerase, ensures proper kinetochore–MT attachment (Holmfeldt *et al*, 2005). An attachment failure leads to missegregation of individual chromosomes.

*MCAK*, located at chromosome 1p34.1, is a member of the kinesin-13 subfamily that shares homology with other members of the kinesin superfamily (Wordeman and Mitchison, 1995; Vale and Fletterick, 1997; Lawrence *et al*, 2004) that play important roles in intracellular transport and cell division (Wittmann *et al*, 2001). Mitotic centromere-associated kinesin is present throughout the cell, but is especially concentrated at the centromeres, kinetochores and spindle poles (Gorbsky, 2004). Although other kinesins act to transport cargo, MCAK and other kinesin-13 members catalyse MT disassembly, an important factor in normal chromosome movement (Helenius *et al*, 2006). Mitotic centromere-associated kinesin destabilises MTs from either end, and this activity and localisation are under the regulation of mitotic kinases (Wordeman *et al*, 2007). Overexpression of wild-type MCAK induces destabilisation of MTs in both mitotic and interphase cells (Kline-Smith and Walczak, 2002).

We recently reported that elevated *MCAK* expression is associated with lymphatic invasion, lymph node metastasis and poor prognosis in gastric cancer (Nakamura *et al*, 2007). Although *MCAK* mRNA is also highly expressed in colorectal cancer tissues (Mori *et al*, 1993a), no clinicopathologic analysis of *MCAK* expression in human colorectal cancer has been reported. In this study, we explored *MCAK* gene expression in human colorectal cancer and evaluated possible associations of gene expression with clinicopathological factors and prognosis.

## MATERIALS AND METHODS

### Cell lines and clinical tissue samples

Cell lines derived from human colorectal cancer (DLD1, COLO201, COLO205, COLO320DM, HT29, HCT15, WiDr, SW480, LS174T, CCK81, RCM1 and CaR1) were obtained from the Cell Resource Centre for Biomedical Research Institute of Development, Aging and Cancer (Tohoku University, Sendai, Japan) and maintained in RPMI-1640 containing 10% fetal bovine serum and antibiotics at 37°C in a 5% humidified CO_2_ atmosphere.

One hundred and twenty patients (70 men, 50 women) with colorectal cancer who underwent surgery at the Medical Institute of Bioregulation at Kyushu University from 1994 to 1999 were included in this study. Primary colorectal cancer specimens and adjacent normal colorectal mucosa were obtained from patients after informed consent had been obtained in accordance with the institutional guidelines of the hospital. Immediately after resection, the necrotic and ulcerated portions of the tumours were removed and normal colonic mucosa was dissociated from muscle and connective tissue. All specimens were immediately frozen in liquid nitrogen and kept at −80°C until RNA extractions were performed. Every patient was definitively identified as having colorectal cancer based on the clinicopathologic findings. None of the patients received chemotherapy or radiotherapy prior to surgery. Clinicopathological factors were assessed according to the criteria of the Japanese Classification of Colorectal Carcinoma (Japanese Society for Cancer Colon and Rectum, 1998). All patients were closely followed after surgery at regular 1-month intervals for 2–72 months, with a mean follow-up period of 38.4 months. This study was conducted under the supervision of the ethical board of Kyushu University.

### RNA preparation and reverse transcription

Total RNA was isolated using a modified acid guanidinium–phenol–chloroform procedure with DNase (Mimori *et al*, 1997). cDNA was synthesised from 2.5 *μ*g of total RNA as described previously (Mori *et al*, 1993b).

### *MCAK* gene amplification

A 237-bp *MCAK* fragment was amplified with the following primers: 5′-GATGGAAGCCTGCTCTAACG-3′ (forward) and 5′-GAGCAGATTCCGCTTTGTTC-3′ (reverse). The forward primer is located in exon 8 and the reverse primer in exon 9. The amplification parameters were 1 min at 95°C, 1 min at 62°C and 1 min at 72°C for 28 cycles. An 8-*μ*l aliquot of each reaction mixture was size-fractionated in a 2% agarose gel and visualised by ethidium bromide staining. To ensure that the RNA was not degraded, a PCR assay with primers specific for the glyceraldehyde-3-phosphate dehydrogenase (*GAPDH*) gene was performed for 1 min at 95°C, 1 min at 56°C and 1 min at 72°C for 28 cycles. The *GAPDH* primers 5′-TTGGTATCGTGGAAGGACTCA-3′ (forward) and 5′-TGTCATCATATTTGGCAGGTT-3′ (reverse) produced a 270-bp amplicon (Ogawa *et al*, 2004). cDNA from the Human Universal Reference Total RNAs (Clontech, Palo Alto, CA, USA) was studied concurrently as a source of positive controls. Confirmation of reverse transcriptase-PCR (RT-PCR) products was performed by direct sequencing analysis.

### Real-time quantitative reverse transcriptase-PCR

Real-time monitoring of the PCRs was performed using the LightCycler FastStart DNA Master SYBR Green I Kit (Roche Diagnostics, Tokyo, Japan). The amplification protocol consisted of 35 cycles of denaturation at 95°C for 10 s, annealing at 64°C for 10 s and elongation at 72°C for 10 s. The products were then subjected to a temperature gradient from 68°C to 95°C at 0.1°C s^−1^ with continuous fluorescence monitoring to produce a melting curve of the products. After proportional background adjustment, the fit point method was used to determine the cycle in which the log-linear signal was distinguished from the background, and that cycle number was used as a crossing-point value. The standard curve was produced by measuring the crossing point of each standard value (twofold serially diluted cDNAs from the Human Universal Reference Total RNAs) and plotting them against the logarithmic value of the concentration. The concentration of each sample was then calculated by setting their crossing points to the standard curve. The expression levels were normalised against *GAPDH* mRNA expression (Ogawa *et al*, 2004).

### Immunohistochemistry

Colorectal cancer surgical specimens from formalin-fixed, paraffin-embedded tissues were used for MCAK immunohistochemistry. After deparaffinisation and blocking, the antigen–antibody reaction was incubated overnight at 4°C. ENVISION reagents (ENVISION+Dual Link/HRP; Dako Cytomation, Glostrup, Denmark) were applied to detect the signal from the antigen–antibody reaction. All sections were counterstained with haematoxylin. The primary anti-MCAK goat polyclonal antibody (ab5966; Abcam, Cambridge, UK) was used at a dilution of 1 : 500.

### Statistical analysis

For continuous variables, the data were expressed as the mean±s.d. The relationship between *MCAK* mRNA expression and the clinicopathological factors was analysed by the *χ*^2^ test and Student's *t*-test. Kaplan–Meier survival curves were plotted and compared with the generalised log rank test. Prognostic factors were evaluated by univariate and multivariate analyses (Cox proportional hazard regression model). A logistic regression model was used to identify the independent predictors of lymph node metastasis. All tests were analysed by JMP software (SAS Institute, Cary, NC, USA), and the findings were considered statistically significant at *P*<0.05.

## RESULTS

### *MCAK* mRNA expression in colorectal cancer cell lines and clinical tissue specimens

**Figure 1:**
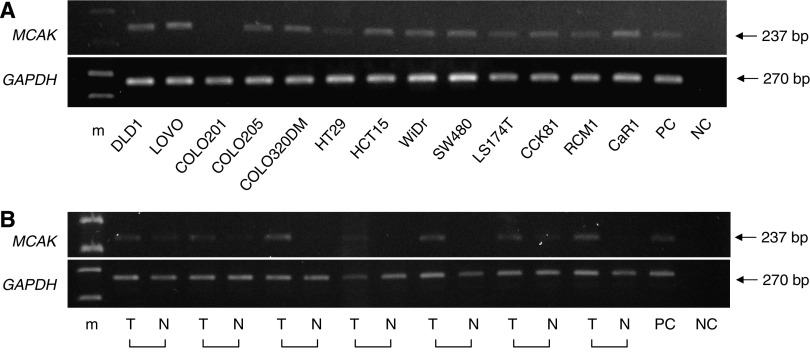
*MCAK* mRNA expression in colorectal cancer cell lines and clinical tissue specimens. (**A**) Reverse transcriptase-PCR analysis of *MCAK* expression in human colorectal cancer cell lines. Twelve of the 13 cell lines (92%) expressed the *MCAK* gene, whereas COLO201 did not. (**B**) Reverse transcriptase-PCR analysis of *MCAK* expression in clinical samples of colorectal cancer. *MCAK* expression was determined in the colorectal tumours (T) and paired normal (N) samples obtained from seven patients. In all seven cases, *MCAK* expression was higher in the tumours than in the normal tissues. *GAPDH* was used as a loading control. m=marker; NC=negative control; PC=positive control.

Figure 1A shows *MCAK* gene expression status in human colorectal cancer cell lines from RT-PCR analysis. Twelve of the 13 cell lines (92%) expressed the *MCAK* gene; COLO201 was the exception. We also performed RT-PCR analysis of *MCAK* in colorectal cancers and paired normal samples obtained from seven patients. In all seven cases, *MCAK* expression was higher in the cancer tissues than in paired normal tissue (Figure 1B). Quantitative real-time RT-PCR was performed using the Human Total RNA Master Panel (Clontech) to characterise *MCAK* mRNA expression. *MCAK* expression was extremely high in the testis. Lower expression was found in the digestive organs (colon and intestines), possibly a reflection of basic mitosis of the mucosa (data not shown). Moreover, quantitative real-time RT-PCR on 120 paired clinical samples showed that 91 of 120 cases (75.8%) exhibited higher levels of *MCAK* mRNA in tumours than in paired normal samples. The mean expression value of *MCAK* mRNA in tumour samples, 0.53±0.037 (mean±s.d., normalised by *GAPDH* gene expression), was significantly higher than the value, 0.32±0.037, for the corresponding normal samples (*P*<0.01; Student's *t*-test).

### Immunohistochemistry

**Figure 2:**
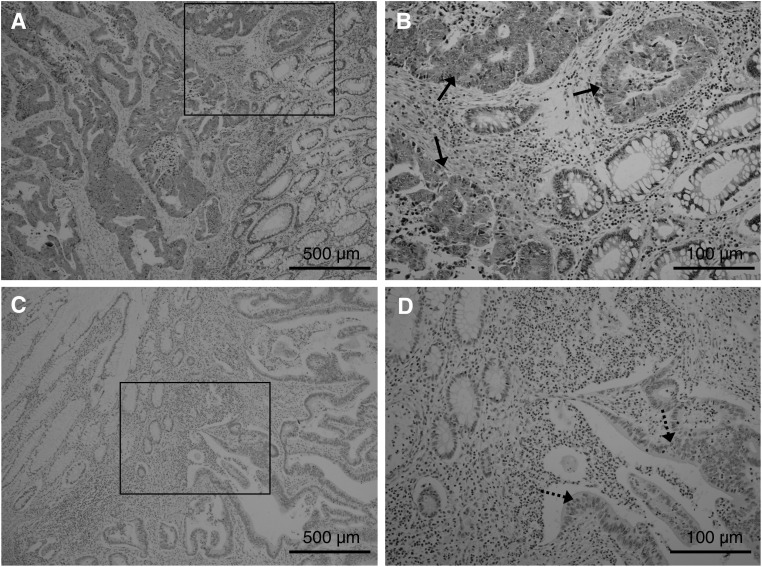
Immunohistochemical staining for MCAK in tumour specimens. (**A** and **B**) A representative positive stain for MCAK in tissue from a colorectal cancer patient. Positive staining is observed in the cytoplasm of cancer cells, but not in the stromal cells nor in normal epithelium. Solid line arrows indicate cancer cells that are positive for MCAK expression. (**C** and **D**) A representative negative stain for MCAK in a colorectal cancer specimen. Dotted line arrows indicate cancer cells that are negative for MCAK expression. Mitotic centromere-associated kinesin protein expression was evaluated in tumours and corresponding normal tissues from 15 representative colorectal cancer cases. The six tumours that were negative for protein expression (**C** and **D**) also exhibited lower mRNA expression levels, whereas seven of nine tumours with a positive immunohistochemical expression (**A** and **B**) displayed elevated mRNA expression levels. The expression of *MCAK* mRNA was thereby associated with protein expression. Each solid square in (**A**) and (**C**) was magnified × 100 and shown in (**B**) and (**D**), respectively (original magnification; (**A**) and (**C**), × 40; (**B**) and (**D**), × 100).

A positive immunohistochemical staining pattern for MCAK in tissue from a colorectal cancer patient is shown in Figure 2A and B. Mitotic centromere-associated kinesin protein staining was observed in the cytoplasm of cancer cells, but not in the stromal cells nor in normal epithelium. Mitotic centromere-associated kinesin protein expression was examined in the tumours and corresponding normal tissues from 15 representative colorectal cancer cases. Of these 15 cases, seven exhibited a higher level of *MCAK* mRNA expression in tumour tissues, whereas the remaining eight cases showed a lower level of *MCAK* mRNA in cancerous tissue. All sections were independently examined for protein expression and scored as positive or negative when >10% (positive) or <10% (negative) of carcinoma cells were stained in an examined area of a specimen. As a result, nine of the representative cases showed a positive reaction for MCAK, whereas six cases were negative. The data were similar to those obtained from *MCAK* mRNA expression analysis. The six negative tumours (Figure 2C and D) also showed lower mRNA expression levels, while seven of the nine positive tumours (Figure 2A and B) showed higher mRNA expression levels. These data suggest that *MCAK* mRNA expression is associated with protein expression.

### *MCAK* mRNA expression and clinicopathological characteristics

The experimental samples were divided into two expression groups. Patients who had less than the median expression ratio of tumour to normal tissue (T/N) were assigned to the low-expression group (*n*=81); the others were assigned to the high-expression group (*n*=39). Clinicopathological factors related to *MCAK* expression status are listed in Table 1. The incidence of three of these factors was positively correlated with increased expression (i.e., the high-expression group): (1) lymph node metastasis was significantly higher (*P*=0.0023) in the high-expression group (25 of 39, 64.1%) than in the low-expression group (28 of 81, 34.6%); (2) the incidence of venous invasion was significantly higher (*P*=0.019) in the high-expression group (13 of 39, 33.3%) than in the low-expression group (12 of 81, 14.8%); and (3) the incidence of peritoneal dissemination was significantly higher in the high-expression group (4 of 39, 10.3%) than in the low-expression group (1 of 81, 1.23%). Moreover, the incidence of advanced stage cancer (according to Dukes' classification) was significantly higher (*P*=0.0023) in the high-expression group (26 of 39, 66.7%) than in the low-expression group (30 of 81, 37.0%). The other clinicopathological factors were not correlated with *MCAK* overexpression.

### Relationship between *MCAK* expression and prognosis

**Figure 3:**
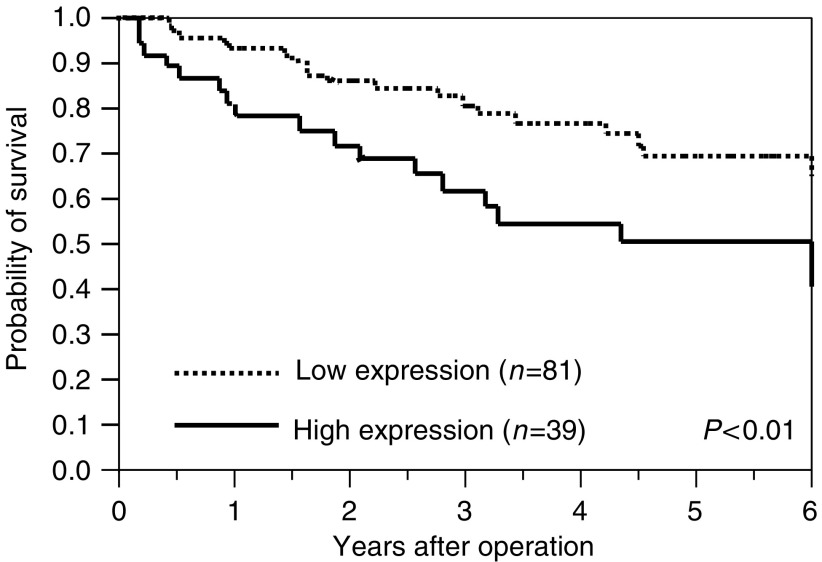
Kaplan–Meier survival curves of patients with colorectal cancer based on *MCAK* mRNA expression status. Patients in the high-expression group (bold line) showed significantly poorer prognosis than those in the low-expression group (dotted line; *P*<0.01, log rank test).

The 5-year survival rate was significantly lower in patients with elevated *MCAK* expression (*P*<0.01; Figure 3). Table 2 provides the univariate and multivariate analyses of factors related to patient prognosis. Univariate analysis showed that the following factors were significantly related to postoperative survival: tumour size (*P*=0.009), serosal invasion (*P*<0.0001), lymph node metastasis (*P*<0.0001), lymphatic invasion (*P*<0.0001) and *MCAK* mRNA expression (*P*=0.019). Multivariate regression analysis indicated that inclusion in the *MCAK* mRNA high-expression group (relative risk (RR), 1.42; 95% confidence interval (CI), 1.01–2.00; *P*<0.05) was an independent predictor of overall survival, as were serosal invasion (RR, 2.16; 95% CI, 1.52–3.12; *P*<0.0001) and lymph node metastasis (RR, 1.82; 95% CI, 1.26–2.76; *P*=0.0012).

### Multivariate analysis for lymph node metastasis

Univariate and multivariate logistic regression analyses were performed on lymph node metastasis (Table 3). Univariate analysis revealed a significant relationship between lymph node metastasis and the following factors: histological grade (*P*=0.04), tumour size (*P*=0.01), depth of invasion (*P*=0.02), lymphatic invasion (*P*<0.0001), venous invasion (*P*=0.003) and *MCAK* mRNA expression (*P*=0.003). Multivariate regression analysis indicated that inclusion in the *MCAK* mRNA high-expression group (RR, 4.02; 95% CI, 1.53–11.3; *P*<0.006) was an independent predictor of lymph node metastasis in addition to lymphatic invasion (RR, 7.67; 95% CI, 3.01–21.1; *P*<0.0001).

## DISCUSSION

In this study, we demonstrated that MCAK is expressed at higher levels in colorectal cancer cells than in the corresponding normal tissues. Mitotic centromere-associated kinesin overexpression causes a moderate increase in the frequency of multipolar spindles (Holmfeldt *et al*, 2004) and monopolar spindles (Kline-Smith and Walczak, 2002), which can contribute to the gain or loss of chromosomes in daughter cells. We reported that overexpression of MCAK leads to the increased migratory and proliferative ability of gastric cancer cells (Nakamura *et al*, 2007). Microtubules, key components of the cytoskeleton, play essential roles in mitosis, cell migration, and cell signalling and trafficking (Honore *et al*, 2005). Mitotic centromere-associated kinesin is present throughout the cell, and overexpression of MCAK may accelerate MT turnover, resulting in the gain of cancer cell motility. This result may partially explain the finding that gastric cancer patients with tumours that express high levels of MCAK had higher rates of lymphatic invasion and metastasis, and a poorer prognosis. Along the same line, downregulation of MCAK in a breast cancer study resulted in tumour growth suppression (Shimo *et al*, 2008). These findings suggest that overexpression of MCAK enhances the malignancy of cancer cells.

Clinicopathological analysis revealed that tumours with high *MCAK* expression were associated with lymph node metastasis, venous invasion, peritoneal dissemination and advanced Dukes' stage. In our previous study on gastric cancer, *MCAK* gene expression in cancer tissues was significantly higher than expression in nonmalignant tissue, and elevated *MCAK* expression was significantly associated with lymphatic invasion and lymph node metastasis (Nakamura *et al*, 2007). These results suggest that MCAK plays a pivotal role in the progression of colorectal cancer and gastric cancer. Moreover, in both studies, patients with high *MCAK* expression had a poorer survival rate than those with low *MCAK* expression, and high *MCAK* expression was an independent prognostic factor for overall survival in the Cox proportional hazard regression model. To our knowledge, this is the first report on correlations between *MCAK* gene expression and clinicopathological factors in colorectal cancer. In the majority of colorectal cancer reports, gene expression is secondary to TNM or Dukes' classification as a prognostic marker. Because its expression is an independent prognostic factor, the expression profile of MCAK may contribute to the creation of a new clinical classification system.

Another aspect of this study is the relationship between *MCAK* expression and lymphatic extension. Interestingly, multivariate analysis for lymph node metastasis revealed that *MCAK* mRNA expression is a good predictor of lymph node metastasis. Lymph node metastases are the most important predictors of survival in non-stage IV colorectal cancer (Schumacher *et al*, 2007). Today, we have several options for curative treatment of early colorectal cancer, such as endoscopic mucosal resection (EMR), endoscopic submucosal dissection (ESD) and laparoscopic-assisted colectomy with regional lymphadenectomy. In limited cases, patients are eligible for such less invasive surgery. Patients suitable for those endoscopic treatments are selected by the preoperative diagnosis of lymph node metastasis, such as macroscopic type, tumour size, presence of an ulcer and histology of biopsy specimens. However, some patients are misdiagnosed and actually do have lymph node metastasis before the operation. By incorporating the genetic diagnosis of *MCAK* gene expression, preoperative selection for patients without lymph node metastasis may be possible. Furthermore, it has been reported that with SELEX, MCAK was detected in the peripheral blood of a colon cancer patient (Scanlan *et al*, 2002), suggesting that the blood level of MCAK can be used for the prediction of lymph node metastasis. The analysis of MCAK using biopsied specimens and blood samples could provide a more accurate evaluation, which might translate to minimally invasive treatments for early colorectal cancer.

In conclusion, the expression of MCAK in colorectal cancer was elevated respective to levels in normal colorectal tissue, and this overexpression was associated with lymph node metastasis, venous invasion, peritoneal dissemination and poor prognosis. In particular, *MCAK* mRNA overexpression was associated with prognosis and lymph node metastasis in a multivariate analysis. Therefore, MCAK could be a useful predictive marker of lymph node metastasis, which might permit minimally invasive and curative treatments combining EMR and ESD for early colorectal cancer.

## Figures and Tables

**Table 1:** Clinicopathological factors and *MCAK* mRNA expression in 120 colorectal cancers

**Factors**	**High expression (%) **(*n*=39)****	**Low expression (%)** **(*n*=81)**	***P*-** **value**
			
Age (mean±s.d.)	65.3±12.3	66.9±11.3	0.49
			
*Gender*			
Male	22 (56.4)	48 (59.3)	0.76
Female	17 (43.6)	33 (40.7)	
			
*Histological grade*			
Well	15 (38.5)	29 (35.8)	0.78
Others (moderately, poorly and mucinous)	24 (61.5)	52 (64.2)	
			


*Tumour size*			
<30 mm (small)	8 (20.5)	17 (21.0)	0.95
⩾30 mm (large)	31 (79.5)	64 (79.0)	
			
*Serosal invasion*			
Absent	26 (66.7)	59 (72.8)	0.49
Present	13 (33.3)	22 (27.2)	
			
*Lymph node metastasis*			
Absent	14 (35.9)	53 (65.4)	0.0023^*^
Present	25 (64.1)	28 (34.6)	
			
*Lymphatic invasion*			
Absent	22 (56.4)	53 (65.4)	0.34
Present	17 (43.6)	28 (34.6)	
			
*Venous invasion*			
Absent	26 (66.7)	69 (85.2)	0.019^*^
Present	13 (33.3)	12 (14.8)	
			
*Liver metastasis*			
Absent	33 (84.6)	73 (90.1)	0.38
Present	6 (15.4)	8 (9.9)	
			
*Peritoneal dissemination*			
Absent	35 (89.7)	80 (98.8)	0.021^*^
Present	4 (10.3)	1 (1.2)	
			
*Dukes' classification*			
A and B	13 (33.3)	51 (63.0)	0.0023^*^
C and D	26 (66.7)	30 (37.0)	

s.d.=standard deviation.

^*^P<0.05.

**Table 2:** Univariate and multivariate analysis for overall survival (Cox proportional hazards regression model)

	**Univariate analysis**			**Multivariate analysis**		
**Factors**	**RR**	**95% CI**	***P-*value**	**RR**	**95% CI**	***P-*value**
Age (<60/60 ⩽)	0.82	0.58–1.20	0.29	—	—	—
Gender (male/female)	0.96	0.68–1.33	0.81	—	—	—
Histological grade (well/moderately, poorly and mucinous)	1.21	0.86–1.76	0.29	—	—	—
Tumour size (<30 mm/⩾30 mm)	1.93	1.16–3.94	0.009	1.29	0.74–2.70	0.4
Serosal invasion (absent/present)	2.40	1.72–3.38	<0.0001	2.16	1.52–3.12	<0.0001
Lymph node metastasis (absent/present)	2.25	1.58–3.38	<0.0001	1.82	1.26–2.76	0.0012
Lymphatic invasion (absent/present)	2.30	1.64–3.31	<0.0001	—	—	—
Venous invasion (absent/present)	1.43	0.99–2.00	0.055	—	—	—
*MCAK* mRNA expression (T/N<3/T/N>3)	1.49	1.07–2.07	0.019	1.42	1.01–2.00	0.048

CI=confidence interval; RR=relative risk.

**Table 3:** Univariate and multivariate analysis for lymph node metastasis (logistic regression model)

	**Univariate analysis**			**Multivariate analysis**		
**Factors**	**RR**	**95% CI**	***P-*value**	**RR**	**95% CI**	***P-*value**
Age (<60/60 ⩽)	0.44	0.18–1.108	0.08	—	—	—
Gender (male/female)	0.86	0.41–1.79	0.69	—	—	—
Histological grade (well/moderately, poorly and mucinous)	2.26	1.05–5.02	0.04	2.21	0.84–6.09	0.11
Tumour size (<30 mm/⩾30 mm)	4.08	1.51–13.1	0.01	2.52	0.72–10.1	0.17
Tumour stage (T1/T2–T4)	4.40	1.34–19.9	0.02	1.25	0.26–7.09	0.79
Lymphatic invasion (absent/present)	9.11	3.98–22.3	<0.0001	7.67	3.01–21.1	<0.0001
Venous invasion (absent/present)	4.41	1.56–8.25	0.003	2.01	0.66–6.48	0.23
*MCAK* mRNA expression (T/N<3/T/N>3)	3.38	1.54–7.67	0.003	4.02	1.53–11.3	0.006

CI=confidence interval; RR=relative risk.
